# Clinical and diagnostic characteristics of Hashimoto’s encephalopathy: a single-center, retrospective study

**DOI:** 10.1007/s13760-024-02520-1

**Published:** 2024-06-11

**Authors:** Jung-Ju Lee, Soo-Min Park, Kyung-Il Park, Kyusik Kang, Woong Woo Lee, Byung Kun Kim, Yong Soo Kim, Ilhan Yoo

**Affiliations:** 1 Department of Neurology, Nowon Eulji Medical Center, Eulji University School of Medicine, 68 Hangeulbisekro, Noweongu, Seoul, 01830 Korea; 2 Department of Neurology, Seoul National University Hospital Healthcare System Gangnam Center, 152 Teheran-Ro, Gangnam-Gu, Seoul, 06236 Korea

**Keywords:** Encephalopathy, Seizure, Thyroid, Antibody, Steroid

## Abstract

**Background and purpose:**

Diagnosing Hashimoto’s encephalopathy (HE) is challenging. In contrast to other types of autoimmune encephalitis, HE shows an excellent response to steroid treatment. We aimed to investigate the rates of antithyroid antibodies (ATAs) and probable HE in patients with unexplained mental dysfunction and compare the clinical characteristics between the good- and poor-outcome groups.

**Methods:**

We retrospectively reviewed the medical records and electroencephalography (EEG) and neuroimaging findings of patients admitted to the Department of Neurology of our hospital from March 1, 2006, to February 28, 2023. Using our proposed diagnostic criteria for probable HE, we compared the clinical characteristics between the good- and poor-outcome groups. We also investigated the rates of ATA positivity and probable HE.

**Results:**

In total, 198 patients exhibited altered mentation, rapidly progressive cognitive decline, or myoclonus. ATA tests were performed on 86 patients, and the detection rates of ATAs and probable HE were 29.1% and 25.6%, respectively. Of the 22 patients enrolled, the good- and poor-outcome groups comprised 19 and 3 patients, respectively. Clinical seizures occurred in seven patients. Nonconvulsive status epilepticus on EEG was observed in six patients, all of whom were intractable to antiepileptic drugs. Nineteen of 21 patients (90.5%) treated with immunosuppressants showed good outcomes.

**Conclusions:**

HE is a rare clinical disorder, but not as rare as previously thought. When HE is suspected, steroids should be considered the first-line treatment. Early diagnosis and adequate treatment are critical to achieve good outcomes in HE.

## Introduction

Hashimoto’s encephalopathy (HE) is a rare neurological disorder that presents challenges in diagnosis. Patients with HE present several symptoms with high levels of antithyroid antibodies (ATAs) [1]. The lack of specific findings in symptoms, neuroimaging, and neurophysiological tests makes it difficult to diagnose HE. Clinical suspicion is the only diagnostic method available, and early diagnosis is critical for treating patients with HE.

Moreover, whether HE is a true existing neurological disorder remains in debate [1–3]. Since the recognition of autoimmune limbic encephalitis (ALE) in the mid-2000s, such as anti-N-methyl-D-aspartate receptor (NMDAR) and leucine-rich glioma-inactivated 1 (LGI1) encephalitis [4], the existence of HE has become more questionable [5]. Some neurologists have asserted that the elevation of ATAs (i.e., thyroid peroxidase antibody [TPOAb] and thyroglobulin antibody [TgAb]) exhibited in HE should be considered an epiphenomenon of other unelucidated or seronegative encephalitis [2, 5, 6].

However, HE has a distinct clinical characteristic other than typical ALEs: patients with HE respond well to treatment with steroids when diagnosed early [7]. ALEs may respond to steroids alone but insufficiently in most cases [8]. Moreover, there have been no consensus diagnostic criteria for HE, which may lead to misdiagnosis or delayed diagnosis of this disorder.

Here, we investigated the rates of ATAs and probable HE in patients with unexplained mental dysfunction and compare the clinical characteristics between the good- and poor-outcome groups. This study aimed to share the 16-year clinical experience at our hospital.

## Methods

### Inclusion of patients

We retrospectively reviewed the medical records and laboratory, neuroimaging, and electroencephalography (EEG) findings of patients admitted with altered mentation, rapidly progressive cognitive decline, or myoclonus of unknown etiology to the Department of Neurology, Nowon Eulji Medical Center from March 1, 2006, to February 28, 2023. The EEG findings were reviewed by two experienced epileptologists.

The modified Rankin Scale (mRS) is routinely used to evaluate all patients admitted to our hospital. Premorbid mRS scores were estimated based on patient history and re-evaluated during the follow-up period.

### Definition of brain lesions

Vasogenic edema (VE) is defined as high signal intensities observed on T2 or fluid-attenuated inversion recovery magnetic resonance imaging (MRI) and apparent diffusion coefficient (ADC) map images without diffusion-weighted imaging (DWI) restriction, cytotoxic edema (CE) with DWI restriction, and low signal intensities on ADC map images. Without these findings, we defined the lesions as old lesions.

### Definition of electroencephalography seizures

EEG seizures are defined as EEG abnormalities compatible with the following: (1) rhythmic or quasirhythmic discharges with spatiotemporal evolution for ≥ 10 s, (2) periodic epileptiform discharges (PEDs) of > 2.5 Hz for ≥ 10 s, or (3) periodic discharges (PDs) with variable periodicity superimposed by rhythmic activity,

### Seropositivity of antithyroid antibodies

Serum levels of TPOAb (normal value: 0–60 U/mL) or TgAb (normal value: 0–60 U/mL) > 150 U/mL were defined as ATA positive.

### Diagnostic criteria for probable Hashimoto’s encephalopathy

Patients who met the following criteria were considered to have probable HE: (1) altered mentation, rapidly progressive cognitive impairments, or myoclonus of otherwise unknown etiology; (2) no symptoms of hyper- or hypothyroidism; (3) no significant elevation or depression of free T4 levels; (4) ATA positivity; (5) no other definite autoimmune disorders; and (6) no evidence of the presence of paraneoplastic antibodies or antibodies associated with ALE. Cerebrospinal fluid examination is not mandatory for diagnosis.

### Patients’ outcomes

Patients were categorized into the good- and poor-outcome groups. A good outcome was defined as (1) an mRS score of ≤ 2, marked improvement of Korean Mini-Mental Status Examination (K-MMSE) score, or returning to premorbid state after treatment and (2) clinical and electrographic seizures ceasing after treatment if patients had seizures. A poor outcome was defined as (1) death, (2) seizures continuing despite treatment, or (3) a follow-up mRS score of ≥ 3 in a patient with a premorbid mRS score of ≤ 2 or a follow-up mRS score that increased in a patient with a premorbid mRS score of ≥ 3.

## Results

**Fig. 1:**
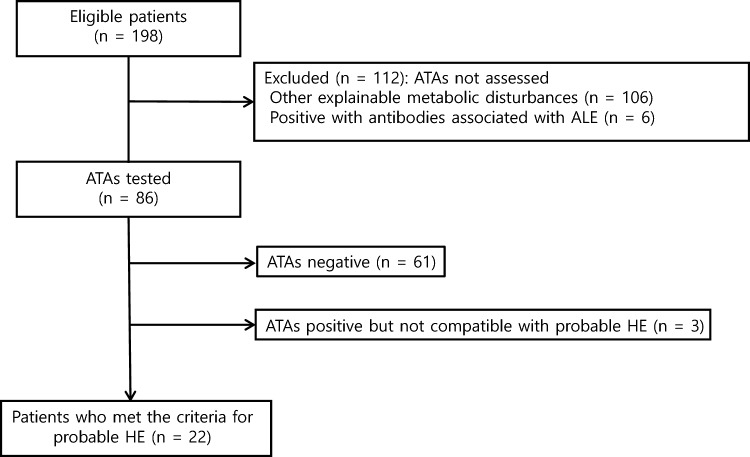
Flowchart of the enrollment of the study participants. Of the 198 patients reviewed for enrollment, 125 were excluded, because their antithyroid antibodies (ATAs) were not assessed as they had other explainable metabolic disturbances (*n* = 122) and positive antibodies associated with autoimmune limbic encephalitis (*n* = 3). In the remaining 73 patients whose ATAs were assessed, 23 were ATA positive. Two patients were ATA positive but not compatible with the criteria for probable Hashimoto’s encephalopathy; thus, 21 patients were finally enrolled. *ATA* antithyroid antibody, *HE* Hashimoto’s encephalopathy

In total, 198 patients exhibited altered mentation, rapidly progressive cognitive decline, and myoclonus. ATA tests were performed in 86 patients, 25 (29.1%) of whom were ATA positive. Tests for diagnosing ALE in both serum and cerebrospinal fluid (CSF) were conducted in 69 patients. Among them, six patients were positively diagnosed with ALE: two patients were attributed to LGI1 encephalitis, one to NMDAR encephalitis, and three to an unknown antibody subtype. Notably, all of the patients with ALE tested negative for antithyroid antibodies (ATAs). Three patients with ATA positivity did not meet the criteria for probable HE: two patients had a history of other metabolic disturbances resolved with the correction of underlying abnormalities (antihistamine overdose and acute kidney injury on chronic kidney disease) and another patient exhibited anti-SOX-1 paraneoplastic antibody. Ultimately, 22 (25.6%) patients were enrolled in this study (Fig. 1). The male-to-female ratio was 3:19, and the mean age was 70.1 ± 9.9 years. Follow-up period was 1 month to 11 years.

The clinical characteristics of the good- and poor-outcome groups are summarized in Tables 1 and 2. Eighteen patients presented with altered mentation, three with progressive cognitive decline (patient 2, 16, and 22), and one with myoclonus alone. Thyroid function tests revealed normal results in 21 of the 22 patients (95.5%). Serum and CSF tests for diagnosing ALE were performed in patients 2, 3, 4, 14, and 21. Solely serum tests were performed in patient 5, 6, 7, 15, 16, 17, and 19. All the patients exhibited negative findings for ALE and paraneoplastic syndromes.

**Table 1:** Good-outcome group

No	Age/sex	Initial symptom	Initial diagnosis	Thyroid function tests	ATAs		Other autoAb	Imaging findings	Initial EEG	Time interval to diagnosis	Treatment/follow-up period	EEG after treatment
					TPOAb (U/mL)	TgAb (U/mL)						
1	F/49	Recurrent amnesia Motionless staring	NCSE	Normal	> 1,300	130.7	ANA (−)	VE: LMTL	Rhythmic theta buildups in the LTL	5 days	AED (CBZ) IV MP Oral PD AZT /10 years	Normal
2	F/54	Psychosis and amnesia followed by seizures	Viral encephalitis	Normal	> 1,300	56.8	ANA (−)	Multiple VE: LTh, RBG, BPL	Normal (interictal period)	3 days	AED (LEV) IV MP Oral PD /8 years	Normal
3	F/73	Fever Motionless staring Stupor	Pneumonia, NCSE	Normal	> 1,300	215.4	ANA (−)	Remnant brainstem glioma	PEDs with rhythmic delta activity in the BFL	10 days	AED (VPA) IV MP Oral PD /6 months	DS
4	F/69	Fever Confusion Aphasia	Viral encephalitis, NCSE	Normal	> 1,300	47.8	ANA (−)	NSA	Rhythmic theta and delta activity with fluctuation in the LFTL	4 days	AEDs (VPA) Oral PD /5 months	Normal
5	M/68	Recurrent sleep attack	OSA or narcolepsy	Normal	< 28	307.7	ANA (−)	NSA	Normal (interictal period)	15 days	Oral PD /3 years	Normal
6	F/72	Stupor	Metabolic encephalopathy, NCSE	Normal	260.7	> 500	ANA (−)	OI: LFTA	Rhythmic delta activity and PEDs in the LFL	5 days	AED (CBZ) IV MP AZT /3 months	FS in the left hemisphere
7	F/74	Confusion with hallucination	Delirium on dementia	Normal	486.7	133.4	ANA (−)	NSA	Normal	7 days	Oral PD AZT /4 months	Normal
8	M/59	Stupor	AKI on CKD	Normal	370.6	63.1	ANA (−)	OH: LBG	DS	8 days	IV MP Oral PD /2 months	FS in the left frontal leads
9	F/78	Confusion	Delirium on dementia	Mild hypothyroidism	411.2	60.6	ANA (−) Anti-ds DNA (−)	Hydrocephalus	FIRDA with DS	10 days	Levothyroxine IV MP Oral PD /3 months	Bifrontal theta slowing
10	F/82	Headache Stupor	SAH	Normal	358.8	31.8	ANA (−)	NSA	Normal (performed after stupor period)	4 days	AZT /6 months	Normal
11	F/61	Confusion Myoclonus	Metabolic encephalopathy, CKD	Normal	64.7	> 500	ANA (−)	NSA	DS	65 days	IV MP Oral PD /4 months	DS
12	F/86	Confusion	AKI on CKD	Normal	392.1	32.7	ANA (−)	OI: RFTA	GTPW	7 days	IV MP Oral PD /3 months	DS
13	F/67	Myoclonus	Cryptogenic	Normal	39.4	> 500	ANA (−)	OI: lacunar infarction	Normal	5 days	AZT /4 months	Normal
14	F/54	Stupor	Septic encephalopathy	Normal	589.6	58.1		AI: lacunar infarction	GTPW	3 days	IV MP Oral PD /3 months	Normal
15	F/71	Confusion	NCSE	Normal	260.7	> 500	ANA (+)	OI: LFTA	Rhythmic theta evolution in the LFL	28 days	AEDs (LEV, VPA) IV MP Oral PD /6 months	FS in the left frontal leads
16	M/77	Progressive cognitive decline	Dementia	Normal	> 1300	> 500		NSA	FIRDA	7 days	IV MP Oral PD AZT /3 months	Normal
17	F/75	Memory disturbance Decreased responsiveness	NCSE	Normal	43.2	185.9		NSA	Rhythmic delta and PEDs in the LFL	5 days	AED (LEV) IV MP Oral PD /8 months	Normal
18	F/65	Hemianopsia Visuospatial dysfunction Language disturbance Confusion	Cerebral infarction	Normal	> 1300	157.6	ANA (−)	AI: LOL	Normal	3 days	IV MP Oral PD /10 months	Normal
19	F/67	Visual disturbance Confusion Irritability	Delirium on dementia	Normal	214.3	< 15.0	ANA (−) Anti-dsDNA (−)	NSA	DS	4 days	IV MP Oral PD /11 years	Normal

*ATA* anth-thyroid antibodies, *TPOAb* thyroperoxidase antibody, *AutoAb* autoimmune antibody, *TgAb* thyroglobulin antibody, *ANA* anti-nuclear antibody, *EEG* electroencephalography, *NCSE* nonconvulsive status epilepticus, *PED* periodic epileptiform discharge, *FIRDA* frontal intermittent rhythmic delta activity, *GTPW* generalized triphasic waves, *VE* vasogenic edema, *AED* antiepileptic drug, *CBZ* carbamazepine, *LEV* levetiracetam, *VPA* valproic acid, *IV* intravenous, *MP* methylprednisolone, *PD* prednisolone, *AZT* azathioprine, *LMTL* left medical temporal lobe, *LTL* left temporal leads, *LTh* left thalamus, *RBG* right basal ganglia, *BPL* bilateral parietal lobe, *BFL* bilateral frontal leads, *LFTA* left frontotemporal area, *LBG* left basal ganglia, *RFTA* right frontotemporal area, *LOL* left occipital lobe, *LFTL* left frontotemporal leads, *LFL* left frontal leads, *DS* diffuse slowing, *FS* focal slowing, *OSA* obstructive sleep apnea, *AKI* acute kidney injury, *CKD* chronic kidney disease, *OI* old infarction, *AI* acute infarction, *OH* old hemorrhage, *NSA* no specific abnormality

**Table 2:** Poor-outcome group

No	Age/sex	Initial symptom	Initial diagnosis	Thyroid function tests	ATA	Other autoAb	Imaging findings	Initial EEG	Time interval to diagnosis	Treatment/follow-up period	EEG after treatment	
					TPOAb	TgAb						
20	F/85	Stupor when found	Acute cerebral infarction, NCSE	Normal	> 1300	131.0	ANA (–)	CE in the BPL	PEDs in the BPOL	3 days	AED (CBZ) /1 month	NC
21	F/74	Intermittent stupor	Metabolic encephalopathy	Normal	351.4	67.2	ANA (–)	NSA	DS	> 90 days	IV MP Oral PD /2 years	NC
22	F/82	Progressive cognitive decline Myoclonus	CJD	Normal	117.4	384.2	ANA (–)	Cortical atrophy	BIPEDs	> 2 years	AED (VPA) IV MP Oral PD /6 months	NC

*ATA* antithyroid antibodies, *TPOAb* thyroperoxidase antibody, *AutoAb* autoimmune antibody, *TgAb* thyroglobulin antibody, *ANA* anti-nuclear antibody, *EEG* electroencephalography, *NCSE* nonconvulsive status epilepticus, *PED* periodic epileptiform discharge, *BIPED* bilateral independent periodic epileptiform discharge, *AED* antiepileptic drug, *CBZ* carbamazepine, *VPA* valproic acid, *IV* intravenous, *MP* methylprednisolone, *PD* prednisolone, *CJD* Creutzfeldt–Jakob disease, *CE* cytotoxic edema, *BP* bilateral parietal lobes, *BPOL* bilateral parietooccipital leads, *NSA* no specific abnormality, *NC* no change, *DS* diffuse slowing

Seizures were observed in seven patients (31.8%): convulsive seizures in one and nonconvulsive status epilepticus (NCSE) in six. PDs, including PEDs and triphasic waves (TPWs), were observed in seven patients, three of whom had EEG seizures. Clinical symptoms and EEG seizures in patients with NCSE did not completely disappear after treatment with antiepileptic drugs (AEDs) alone but disappeared after treatment with immunosuppressants. AEDs were not administered to patients with TPWs. After treatment with steroids, the TPWs disappeared. AEDs were administered to patients with PEDs, but clinical symptoms and EEG abnormalities persisted.

VEs were observed in two patients, which disappeared after treatment. CEs were observed in one patient (patient 19) who exhibited PEDs. Acute infarction (AI) was observed in two patients. The AI size in both patients was small; patient 14 had a lacunar infarction in the left basis pontis, and patient 18 had tiny dot-like lesions in the left occipital lobe. Patient 18 initially presented with visual disturbance and hemianopsia but progressed to language disturbance and confusion. The lesions did not extend on the follow-up DWI. Old focal lesions were observed in six patients.

Twenty-one patients were treated with immunosuppressants: oral or parenteral steroids in 19, azathioprine (AZT) in six, and both in four patients. AZT alone was administered to patients with steroid contraindications. Among the patients treated with immunosuppressants, 19 (90.5%) showed good outcomes. In the poor-outcome group, two patients were treated with immunosuppressants. The diagnostic delays were > 3 months (> 3 months in patient 21, > 2 years in patient 22). Although the diagnostic delay was relative short, patient 20 was not treated with immunosuppressants and showed poor outcome.

Patients 8, 11, 12, and 14 had other accompanying metabolic disturbances, and patients 12 and 14 exhibited TPWs. However, after controlling for metabolic disturbances, the symptoms did not completely resolve. After the detection of ATA and steroid treatment, the patients showed good outcomes. TPWs disappeared after steroid treatment.

Out of the 16 patients with abnormal EEGs, 13 individuals exhibited significant electrographic improvement following the treatment. Six of these patients achieved normalization. Conversely, the three patients in a poor-outcome group did not demonstrate any electrographic improvement.

Patient 2 and 16 showed marked improvement of K-MMSE score after treatment: 10 to 26 in patient 2, and 12 to 18 in patient 16. Patient 2 recovered fully to premorbid state during admission period. Patient 16 improved much after 3-month treatment with immunosuppressants but has been lost to follow-up since then.

Patient 22 failed to show any improvement after treatment (0 to 0 on K-MMSE). The patient was previously diagnosed with Creutzfeldt–Jakob disease (CJD) at another hospital because of the rapid progression of cognitive decline for a few months at that time. However, the clinical state of the patient has progressed slowly for more than 2 years. The patient was mute but alert and responded to pain and a loud voice upon admission. The patient’s serum TgAb levels were high, and steroids with valproic acid were administered but failed to improve the patient’s state.

## Discussion

Since the first report in 1966 [9], the existence of HE has been in debate. The elevation of ATA levels is often observed in several other illnesses, such as psychiatric, degenerative, and other immunological disorders [10, 11]. Other immunological disorders respond relatively well to treatment with immunosuppressants [12]. However, HE dramatically responds to steroids and other immunosuppressants [1, 2, 7], which is a distinctive feature of this disorder. In our study, 90.5% of the patients treated with steroids and AZT showed good outcomes. Patients who had not been treated with steroids and had diagnostic delays more than 3 months showed poor outcomes.

Myxedematous coma is characterized by severe hypothyroidism causing decreased mental status, hypothermia, and other symptoms related to slowing of function in multiple organs [13]. Coma in thyroid storm is characterized by an extreme, decompensated, life-threatening state of thyrotoxicosis [14]. These two disorders are caused by shortage or excess of thyroid hormone. The pathophysiology of HE is not fully understood; however, it is considered an autoimmune and not a hormonal disorder [1–3, 7].

Similar to our study, normal thyroid function or subclinical thyroid dysfunction has been reported, rather than overt thyroid dysfunction [7, 15, 16], for which the diagnostic difficulty lies. Clinical suspicion is key to diagnosis. If no other explainable cause is found on routine laboratory tests and neuroimaging, ATA levels should be assessed. Moreover, when treatment of an underlying metabolic disturbance fails to improve a patient’s mental status, although laboratory and EEG findings are compatible with metabolic disturbances, such as uremic or septic encephalopathy (as shown in patients 8, 11, 12, and 14), ATA levels should be assessed to exclude the possibility of HE. In this study, the detection rates of ATAs and probable HE were 29.1% and 25.6%, respectively.

Convulsive seizures and NCSE were observed in seven patients. HE is a cause of intractable status epilepticus [17]. The NCSE presented in our patients may have contributed to altered mentation or cognitive decline but was uncontrollable with AEDs alone. AEDs and immunosuppressants should be co-administered to such patients.

PDs incompatible with EEG seizures were observed in four patients; AEDs were administered to two patients who showed PEDs but failed to show any clinical effect. PEDs may reflect seizure activity and other acute structural cerebral disorders [18–20]. Several etiologies, including CJD, have demonstrated PEDs [21]. PEDs may also emerge after the cessation of prolonged seizures [22]. Patient 20 may have had unobserved prolonged seizures during sleep. PEDs with DWI restriction may provide evidence of prolonged seizures [23]. Poor patient outcomes may be related to the severity of the disorder, lack of treatment with immunosuppressants, or both. HE may mimic the clinical symptoms of CJD [24]. CJD is a fatal disorder without specific treatment and is characterized by progressive cognitive decline; involuntary movement, including myoclonus; and eventual progression to death within a year. The first two signs and symptoms described above are also common in HE [2, 7]. However, the prognoses of these two disorders significantly differ. Elevated 14-3-3 protein, which is considered a hallmark of CJD, also has been reported in a patient with HE [25]. When typical imaging findings of CJD are absent, HE should be excluded first.

Vasculitis is a proposed mechanism for HE [26]. An autopsy case of HE demonstrated lymphocytic vasculitis of the veins and venules of the brain stem [27]. VEs in our patients may be associated with vasculitis. VEs on neuroimaging are common findings of vasculitis, and VEs observed in vasculitis respond well to steroids [28]. Other patients without VEs on MRI may also have vasculitis pathology, because most of them showed good response to steroids.

AIs were observed in two patients. AIs might be a coincidental finding, play a role as a triggering factor, or have been caused by the vasculitic pathogenesis of HE [29]. Initial symptoms of patient 18 were compatible with the neuroimaging findings, but other cognitive symptoms that are incompatible with lesions occurred subsequently. Further investigations are necessary to elucidate the relationship between AI and HE.

Of the 198 patients, six patients were diagnosed with ALE and one patient with paraneoplastic encephalitis. In patients with ALE, the ATA test result was negative. In a patient with paraneoplastic encephalitis who tested positive for the SOX-1 antibody, ATA level was elevated. Although ALE and paraneoplastic encephalitis are rarer than HE, they should be excluded. A previous report indicated that ATAs might be elevated in patients with ALE or paraneoplastic disorder [5]. Other autoimmune encephalopathies were not definitively ruled out in all subjects. Antinuclear antibodies were assessed in 19 individuals, while anti-double-stranded DNA antibodies were examined in two cases. Since some patients within our study were enrolled prior to the identification of ALE, serum and CSF analyses for ALE were not uniformly performed in all subjects diagnosed with HE. However, steroid response of those disorders is poorer than that of HE, which is the differential point from ALE and paraneoplastic encephalitis.

In conclusion, HE is a rare clinical disorder. However, with clinical suspicion, it is not as rare as previously thought. In this study, the detection rate of probable HE was as high as 25.6%. Therefore, HE may have been underdiagnosed in clinical practice. HE should be considered in the differential diagnosis, even in patients with underlying metabolic disturbances, especially when treatment of these disturbances fails to improve mental status. When HE is suspected, steroids should be considered the first-line treatment. Azathioprine is an alternative treatment for patients in whom steroids are contraindicated. Timely and adequate management is critical to improve the outcomes of patients with HE.

## Data Availability

Data openly available in a public repository that issues datasets with DOIs.
